# Using ante-natal clinic prevalence data to monitor temporal changes in malaria incidence in a humanitarian setting in the Democratic Republic of Congo

**DOI:** 10.1186/s12936-018-2460-9

**Published:** 2018-08-29

**Authors:** Joel Hellewell, Patrick Walker, Azra Ghani, Bhargavi Rao, Thomas S. Churcher

**Affiliations:** 1 MRC Centre for Outbreak Analysis and Modelling, Imperial College London, London, UK; 2 Manson Unit, Médecins Sans Frontières (Operational Centre Amsterdam), London, UK

**Keywords:** *Plasmodium falciparum*, Malaria in pregnancy, Epidemiology

## Abstract

**Background:**

The number of clinical cases of malaria is often recorded in resource constrained or conflict settings as a proxy for disease burden. Interpreting case count data in areas of humanitarian need is challenging due to uncertainties in population size caused by security concerns, resource constraints and population movement. Malaria prevalence in women visiting ante-natal care (ANC) clinics has the potential to be an easier and more accurate metric for malaria surveillance that is unbiased by population size if malaria testing is routinely conducted irrespective of symptoms.

**Methods:**

A suite of distributed lag non-linear models was fitted to clinical incidence time-series data in children under 5 years and ANC prevalence data from health centres run by Médecins Sans Frontières in the Democratic Republic of Congo, which implement routine intermittent screening and treatment alongside intermittent preventative treatment in pregnancy. These statistical models enable the temporal relationship between the two metrics to be disentangled.

**Results:**

There was a strong relationship between the ANC prevalence and clinical incidence suggesting that both can be used to describe current malaria endemicity. There was no evidence that ANC prevalence could predict future clinical incidence, though a change in clinical incidence was shown to influence ANC prevalence up to 3 months into the future.

**Conclusions:**

The results indicate that ANC prevalence may be a suitable metric for retrospective evaluations of the impact of malaria interventions and is a useful method for evaluating long-term malaria trends in resource constrained settings.

**Electronic supplementary material:**

The online version of this article (10.1186/s12936-018-2460-9) contains supplementary material, which is available to authorized users.

## Background

Malaria remains endemic across large portions of the world, with an estimated 216 million clinical cases and 445,000 deaths globally during 2016 [1]. This burden falls disproportionately on young children in countries where the climate is amenable to endemic malaria transmission [2], predominantly sub-Saharan Africa. The increased investment in malaria treatment and prevention, along with the diverse methods available for malaria control, makes the effective measuring of temporal trends in malaria burden critically important [3]. The effectiveness of control interventions varies from site to site due to the epidemiology of infection and factors, such as the susceptibility of the local mosquito population to insecticides [4]. Local control programmes need to monitor the impact of interventions to identify the optimum package, justify future financial investment, and identify changes in transmission in a timely manner [5].

Africa-wide estimates of burden reduction have primarily utilized cross-sectional survey data conducted by the Demographic and Health Surveys Programme [6, 7]. These surveys are undertaken at the province level, usually every 2–3 years, where children are tested for malaria in randomly selected clusters. Province-wide estimates can hide substantial spatial heterogeneity generated by local healthcare provision or local geographical, demographic or climatic differences, therefore, populations in some areas face higher malaria burdens than the province-wide average [8, 9]. Finer scale estimates of burden can be collated passively using the number of malaria cases reported from local health centres. To generate meaningful incidence rates requires good estimates of the size of the health catchment population, which is unlikely to be available in many parts of sub-Saharan Africa. The problems are exaggerated in humanitarian settings where populations may be highly transient, or size estimates hard to generate due to security concerns or resource constrains. This is especially the case in ‘open’ chronic conflict settings where displaced populations often live amongst the local population and not in a defined enclosed area or are frequently on the move due to insecurity. The prevalence of the malaria parasite in refugee and internally displaced populations is often higher than in local more stable populations due to inequalities in resources and health provision [10].

**Fig. 1:**
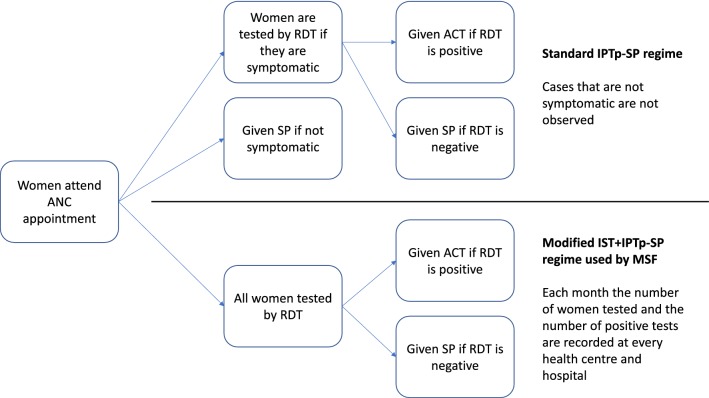
Flowchart illustrating the difference between the standard intermittent preventative treatment during pregnancy using sulfadoxine-pyrimethamine (IPTp-SP) regimen and the expanded intermittent screen and treat plus IPTp-SP (IST+IPTp-SP) regime used by MSF in their ANC programmes in malaria endemic countries

A novel method for routine malaria surveillance could be the use of ante-natal care (ANC) data [11]. Such data are used in sentinel surveillance surveys for HIV, as it corresponds well with national HIV survey data of the same catchment areas [12]. For malaria, the prevalence of infection in pregnant women is strongly correlated with the prevalence of infection in children under 5 in cross-sectional survey data from across Africa [13]. During standard intermittent preventative treatment during pregnancy (IPTp) programmes, any woman that is symptomatic is tested by RDT and given artemisinin-based combination therapy (ACT), if they test positive. Any women who are not symptomatic or are test-negative are given chemoprevention in the form of sulfadoxine-pyrimethamine (SP). Since 2011, Médecins Sans Frontières (MSF) has rolled out a model of routine intermittent screening and treatment (IST) of all pregnant women combined with the IPTp-SP programme described above. This entails testing all pregnant women at every ANC appointment, women who are test-positive are given ACT and women who are test-negative are given SP (Fig. 1).

Since all women are tested regardless of symptoms, this reduces under-reporting bias due to the presence of asymptomatic infections. ANC programmes run by MSF in malaria endemic countries record the number of RDTs administered and the number of positive test results during ANC appointments at each health facility or hospital every month.

Here, methods are developed to predict the relationship between the prevalence of infection in pregnant women and the clinical incidence in children under 5 years old, using field data collected at five MSF field sites in the Democratic Republic of Congo (DRC). There is population denominator data available at these five field sites, which is uncommon for many of the sites where MSF works and more widely across sub-Saharan Africa. Nested statistical models are used to investigate the relationship between ANC prevalence and clinical incidence and determine whether this association is immediate or spread out over time. The utility of routinely collected ANC data for malaria surveillance and the evaluation of control interventions is then discussed, with special regard for settings where such denominator data are not available.

## Methods

The data comprises time series from 5 different MSF health centres across the DRC for varying amounts of time between 2010 and 2016. These MSF missions vary in size and represent a mixture of hospitals, health centres and community clinics in the Great Lakes region; from North and South Kivu, close to the eastern border with Rwanda and Burundi (Baraka, Kimbi-Lulimba, Mweso and Walikale) and from the South-East province of Katanga, bordering Tanzania and Zambia (Shamwana, closed by the end of 2016). All sites are considered ‘open’ humanitarian settings, i.e. areas of chronic conflict mainly from the ongoing Congolese civil war, including internally displaced peoples (IDPs) and with frequent population movement due to fighting.

The ANC prevalence time series is the number of pregnant women tested for malaria using RDTs and the proportion of these that tested positive. Data is collated each month and all women that attend ANC appointments are tested for malaria regardless of whether they are symptomatic. The second time series is the monthly clinical incidence in children under 5 confirmed by RDT (i.e. symptomatic cases arriving as outpatients that tested positive by RDT). The size of the under 5 population at Mweso, Walikale and Shamwana is estimated by MSF each month using population surveys. The size of the under 5 population at Baraka and Kimbi-Lulimba, which cover larger areas, is taken from national census data conducted during the period of investigation by the DRC Department of Health.

**Fig. 2:**
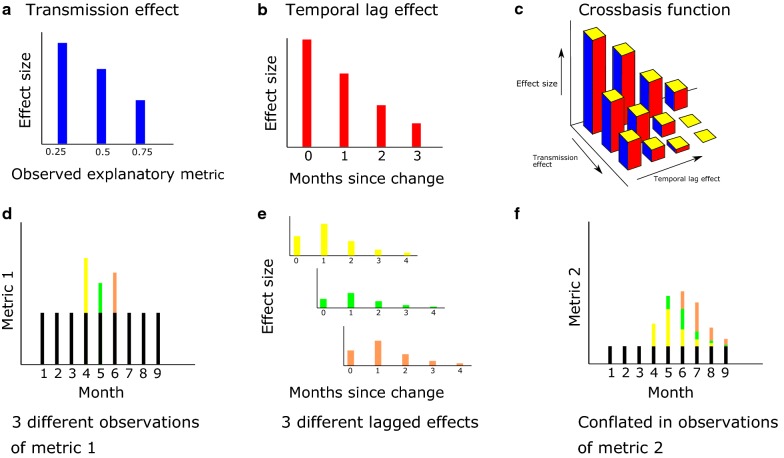
**a**–**c** The concept of a crossbasis function in this context, in **a** the explanatory metric has corresponding effect on the response metric, the function that explains this relationship is the transmission effect basis. In **b** for a given value of the explanatory metric, this may have delayed effects on the response metric—in this plot for 3 months afterwards. This relationship is characterized by the temporal lag basis. In **c**, these two basis functions are combined into a bi-dimensional plot, the shape of the crossbasis function is restricted by the choice of functions in **a** and **b**. The precise shape of the crossbasis is determined during the fitting of the DLNM model. **d**–**f** How subsequent changes in one metric (Metric 1) can cause unpredictable patterns in another metric (Metric 2). **d** The different changes in Metric 1 differentiated by colour (yellow for the change in month 4, green for the change in month 5 and brown for month 6). **e** Each of these changes in Metric 1 have lagged effect that may differ with the size of the observation in Metric 1 and start at different times. These lagged effects are then observed as changes in Metric (2) over multiple months (**f**) with the lagged effects of three different changes in Metric 1 stacking up to create complex patterns in Metric 2. This is illustrated in this example where month 4 saw the greatest increase in Metric 1 whilst Metric 2 peaked in month 6

An illustration of how the change in one metric may continue to influence another metric in the future (a lagged effect) is shown in Fig. 2. If one metric can affect another second metric for a long period of time, then the value of the second metric will depend on the current and historical values of the first metric.

A causal framework was utilized to characterize the relationship between ANC prevalence and clinical incidence, as well as to determine the direction of the association between the two metrics. A variable *X* “Granger causes” *Y* if including past values of *X* in a predictive model of *Y* produces better predictions of *Y* than just using past values of *Y* alone [14]. The analysis follows a two-step process. Firstly, a Granger causality test is used to determine the direction of the association (whether changes in ANC prevalence can predict future changes in clinical incidence, or vice versa) as well as the duration of any lagged effect. Secondly, this relationship is then fully characterized using more complex statistical models to determine the magnitude of the lagged effects and how the association might change with disease endemicity.

A vector auto-regression (VAR) model is used to test for Granger causality between the two metrics, determining the direction and length of potential lagged effects between two or more time series [15]. Granger causality was tested for using a Wald test suitable for stationary time series [16]. The number of past observations that should be used in the VAR model (known as the lag order) is determined by finding the lag order that optimizes some information criterion, usually the Akaike information criterion [17]. The VAR model with the optimum lag order was assessed for goodness of fit by examining the model residuals, performing a multivariate Portmanteau test to confirm that they are not correlated with each other and an autoregressive conditional heteroscedasticity test that looks for changing variance over time. The VAR models were fit using the package ‘vars’ in the R statistical software [16].

Distributed lag non-linear models (DLNMs) are used to fully characterize the relationship between the two metrics, these flexible models allow a “lagged effect” as well as an “endemicity effect” of one metric upon the other. The “lagged effect” means that the effect of the explanatory metric upon the response metric happens over time (with the effect size changing with respect to time), whereas the “endemicity effect” enables the relationship between the two metrics to change according to the level of disease (the effect size varies with the value of the explanatory metric) [18]. DLNMs are specified by choosing two “basis” functions, the first basis function describes the shape of the association between the two metrics at each point in time (the transmission effect basis), the second basis function controls the shape of the lagged effects in the model (the temporal lag basis, an example being Fig. 2b). These two functions are combined into a “crossbasis” function that describes the relationship between the value of an observation, how long ago it was observed and what its current effect will be on the response variable [19]. The crossbasis function can vary in shape depending on the two individual functions used to construct it. A crossbasis function can be written as *s*(*x*_*t*−*l*_, *t *− *l*; *η*), where *x*_*t*−*l*_ is the observation of the explanatory variable *l* months ago, *t*−*l* is the number of months since the observation, and *η* are the so-called “basis parameters” which are the parameters that describe the shape of the two functions combined in the crossbasis. The crossbasis function can be included as a predictor in a generalized additive model with the following form:

1 $$logit\left( {E\left( {Y_{t} } \right)} \right) = \alpha + h_{i} + \sum\nolimits_{l = 0}^{L} {s\left( {x_{t - l} ,\,\,t - l;\eta } \right)} ,$$

where *E*(*Y*_*t*_) is the expected value of the response variable at time *t* (as determined by the Granger causality test outlined above), *x*_*t*−*l*_ is the value of the explanatory variable at time *t *− *l*, *α* is a parameter determining mean difference between the two metrics, *h*_*i*_ is the location-specific modifier of the mean difference between the metrics for location *i,* and *L* is the optimal lag order found when fitting the VAR model (and takes a value of 0 in models with no lagged effects). Different crossbasis functions (*s*(*x*_*t*−*l*_, *t* − *l*; *η*)) made up of the two different basis functions are fit to the observed data and compared to determine the most parsimonious model. Two different functions are used to investigate how the relationship between metrics changes with endemicity, i.e. the transmission effect basis:

- Linear basis: The simplest model assumes that the endemicity effect varies linearly with the explanatory metric.
- Hill function: A function flexible enough to fit the relationship between the incidence and prevalence typically observed in non-temporal data [20].

A choice of three different basis functions are used as the temporal lag basis:

- No lagged effect.
- Linear basis: The effect of a change in the explanatory metric increases or decreases linearly with respect to time.
- Non-linear basis: A non-linear spline function that is penalized to produce a smooth curve, using penalized splines has been shown in simulations to be an effective method of reconstructing a variety of lag-exposure relationships when fitting DLNMs [21].

All combinations of endemicity effect and lagged effect basis functions are tested, giving a total of six different models. For clarity, each model is named with an acronym that represents its structure. The first two letters of the acronym represent the function used for the transmission effect basis, this can be either LE for a linear function or NE for a Hill function. The second two letters indicate the function used for the temporal lag basis, this can be LL for linear lagged effects or NL for non-linear basis spline lagged effects. If there is only one pair of letters then the model does not have lagged effects. The names of all six models are listed in Table 2.

Models were fit using the ‘*dlnm*’ package [22] for the R statistical software and the most parsimonious model was identified using AIC value. The predictive power of each model (its ability to correctly predict into the future) was compared use a rolling origin cross-validation method. This predicted a year of unseen data at a time, with the model being fit using all previous years of data at the given location and all the data from every other location. The models can then be compared using the root mean squared error of their predictions.

## Results

**Fig. 3:**
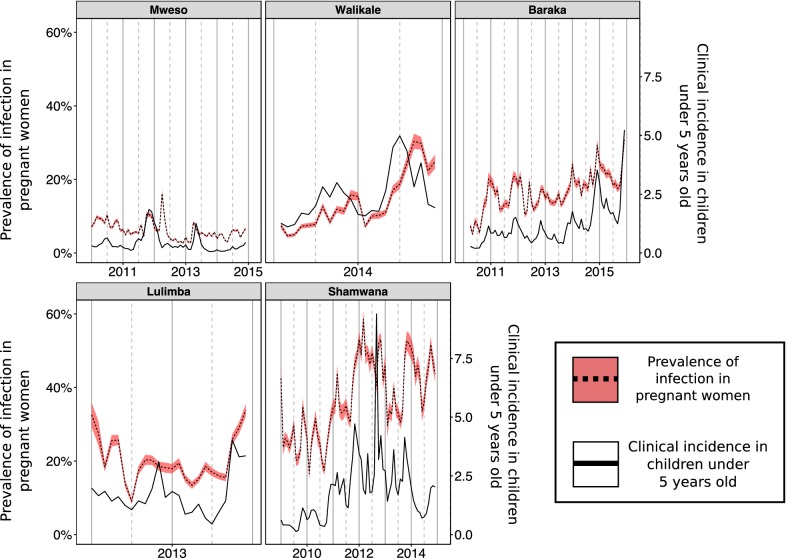
Time series data from the five different settings used in the analyses. The solid black line shows the recorded clinical incidence rate in children under 5 years old each month (cases per child per year). The dotted black line shows the recorded anti-natal clinic prevalence recorded each month with the red shaded area indicating the 95% confidence intervals using the normal approximation method. Data are available for different durations in the different settings

ANC prevalence and clinical incidence in children under 5 across the five locations are shown in Fig. 3. Visually, it is clear that the temporal trends in the metrics are broadly the same, though the association has substantial variability over time and between different locations. Baraka and Shamwana show pronounced seasonal patterns in both transmission metrics, whereas the other sites do not show obvious seasonal variation in transmission. In Fig. 3 the sites are ordered from the northernmost site to the southernmost site when moving from left to right along the top row and then the bottom row, there is a steep gradient in the degree of seasonality of malaria transmission when moving from north to south [23].

**Fig. 4:**
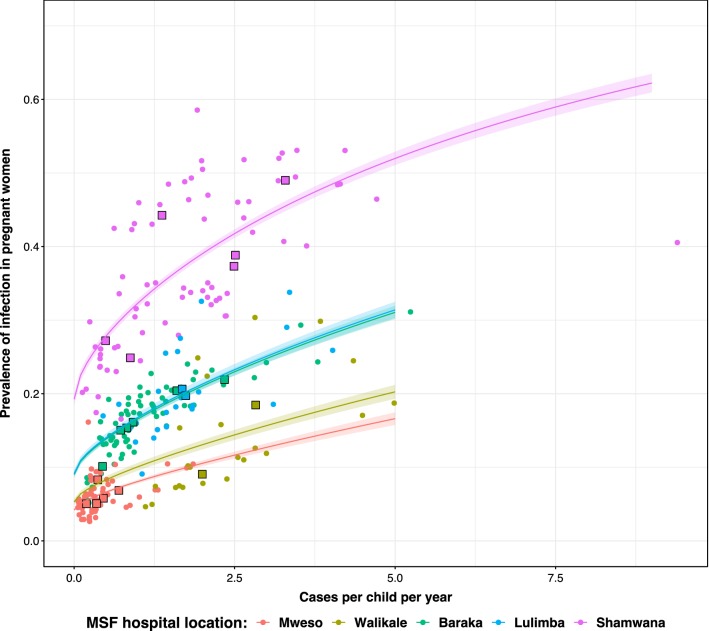
Cross-sectional relationship between prevalence of infection in pregnant women attending anti-natal clinics (ANC) and clinical incidence in children under 5 years reported at the same site. The small circular points show the raw monthly values, coloured by location. The large square points show the same data aggregated by calendar year. The coloured curves show a simple non-linear relationship between the two metrics with no lagged effects (equivalent to model NE) and corresponding 95% confidence interval

Different sites also have differing levels of ANC prevalence despite similar incidence rates in children under 5. For example, Shamwana and Kimbi-Lulimba have median observed clinical incidence rates in children under 5 of 1.714 and 1.711 respectively, but their median observed ANC prevalence is 34.6% in Shamwana and 18.5% in Kimbi-Lulimba (Table 1). A direct cross-sectional comparison of the two metrics each month is shown in Fig. 4.

**Table 1:** Summary of the time series data collected during the same month from the different DRC settings

Location	Number of data points in time-series	Median population size	Median monthly ANC visits	Median monthly ANC prevalence (%)	Median incidence in children under 5 years (minimum, maximum)
Baraka	69	71,238	636	17.3	0.929 (0.199, 5.24)
Mweso	60	65,867	1074	5.7	0.277 (0.059, 1.854)
Walikale	23	31,536	437	11.3	2.072 (1.112, 4.986)
Shamwana	72	36,000	455	34.6	1.714 (0.129, 9.397)
Kimbi-Lulimba	24	15,812	582	18.5	1.711 (0.451, 4.028)

The population size of the catchment area (used to convert case numbers into clinical incidence rates are and the number of women attending anti-natal clinics (ANC visits) are summarized using the median value. The longitudinal time series is shown graphically in Fig. 3

The Granger causality test indicated that past clinical incidence can significantly improve predictions of future ANC prevalence compared to past values of ANC prevalence alone (*p *= 0.002). Conversely, ANC prevalence was unable to predict future clinical incidence with significantly more accuracy compared to using past values of clinical incidence alone (*p *= 0.42). The subsequent analysis therefore uses clinical incidence in children under 5 years as the explanatory variable and ANC prevalence as the response variable. The VAR model used for Granger causality testing also determined the length of the lagged effect (how many previous months of clinical incidence in under 5 s are predictive of the current ANC prevalence), the VAR model with the optimum AIC value had a maximum lag value of 3 months (1 month AIC = − 6.544, 2 months AIC = − 6.556, 3 months AIC = − 6.581, 4 months AIC = − 6.574). Since the difference in AIC values between the models with different lag values was not large enough to decisively prefer one model, the later DLNM model NENL was also fit using maximum lag values of 1, 2 and 4 months (see Additional file 1).

**Fig. 5:**
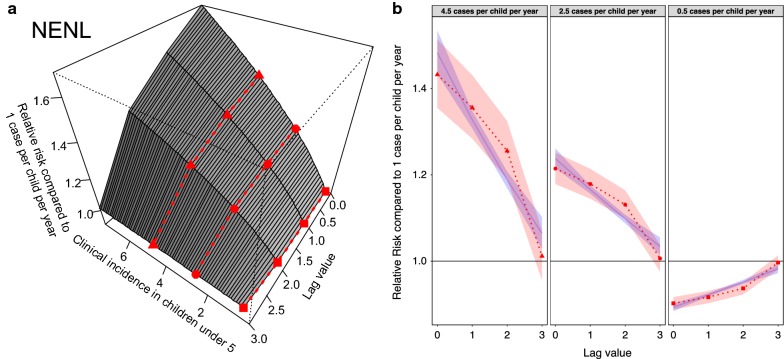
The best fit “NENL” model showing how clinical incidence over the last 3 months influences current anti-natal clinic (ANC) prevalence in terms of relative risk when compared to an observation of 1 case per child per year. **a** Gives the full 3D relationship (the crossbasis function). Values greater than 1 indicate an increase in ANC prevalence whilst values less than one signify a decline. **b** Cross-sectional slices through the crossbasis function at three different clinical incidence values denoted by the shape of the points and corresponding lines on **a**. The red shaded band shows the 95% confidence interval in the fitted lagged effects whilst the blue line and associated band show the lagged effects predicted by the model NELL (allowing a comparison between the linear and non-linear lagged effects)

The “NENL” model provides the best fit (in terms of both AIC value and out-of-sample predictive power) indicating that changes in clinical incidence impact ANC prevalence non-linearly according to the level of endemicity, and that these effects manifest themselves (again non-linearly) immediately and over the subsequent months (Table 2). The 3D relationship (crossbasis function) is shown in Fig. 5a whilst a representation of the temporal lag basis function is depicted for various endemicity levels in Fig. 5b. The lagged effects are significant for 3 months, with the effect size being greatest in the month that the change in incidence is observed and then decreasing over time. The best fitting model that uses non-linear splines to model lagged effects (NENL) is an improvement, albeit a smaller one, upon the similar model that uses a linear function to model lagged effects (NELL). The non-linear lagged effects (NENL) estimate that incidence has a bigger effect on ANC prevalence with 1 and 2 months lag than the linear model (NELL) predicts (Fig. 5b).

**Table 2:** Summary of the different distributed lag non-linear models (DLNMs) characterizing the relationship between clinical incidence and ante natal clinic (ANC) parasite prevalence

Acronym	Endemicity effect	Lagged effect	Number of parameters	AIC	RMSE (rolling cross-validation)
LE	Linear	No lagged effects	6	3859.2	0.0667
LELL	Linear	Linear	7	3116.6	0.0563
LENL	Linear	Non-linear	13	3116.0	0.0564
NE	Hill function	No lagged effects	8	3499.8	0.1126
NELL	Hill function	Linear	9	2982.0	0.05434
NENL	Hill function	Non-linear	15	*2978.9*	*0.05431*

The second and third columns indicate the shape of the basis function used to characterize how the relationship is influenced by endemicity and the lagged effect. Models are compared using Akaike information criterion (AIC, lowest value in italic indicating most parsimonious model) and root mean squared error (RMSE, lowest value in italic indicating most predictive model)

**Fig. 6:**
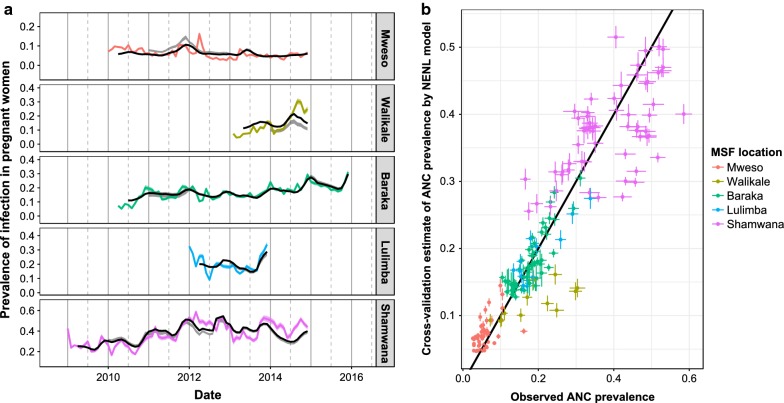
The results of the out-of-sample prediction for the best fitting “NENL” model. This uses at least one previous year of data as a training dataset before trying to make out-of-sample predictions for the subsequent years. **a** The coloured lines show the observed ANC prevalence each month at each location and their corresponding 95% confidence interval. The black line shows the model predictions of the ANC prevalence when the model was fit using all data. The grey band shows a 95% confidence interval for the rolling origin cross validation technique. **b** Points show a comparison of observed ANC prevalence and the corresponding out-of-sample predictions, coloured by site. Lines around the points show the 95% confidence interval for the observations and out-of-sample prediction. The black line shows a perfect correspondence between observation and prediction

Allowing the relationship between clinical incidence and ANC prevalence to be non-linear substantially improves model fit (Table 2). A graphical representation of the out-of-sample predictive power of the best “NENL” model is shown in Fig. 6. Though the best-fit model is unable to predict small changes in prevalence the overall trends are well captured. How well the model captures trends in prevalence is demonstrated both when the model is fit to all available data and when using the rolling origin cross validation technique, where predictions are made using the history of infection from the last year or more.

## Discussion

Clinical incidence in children under 5 years old could predict ANC prevalence but not vice versa. This matches our current understanding of the epidemiology of malaria. Clinical incidence in children under 5 years, who have low levels of malaria immunity, is likely to closely reflect the incidence of new infections and thus be a good proxy for the current intensity of transmission. Conversely, in pregnant women an infection, and associated HRP-2 antigenaemia, can persist asymptomatically for a prolonged period of time. Since pregnant women are being tested routinely, regardless of symptoms, ANC-based prevalence is likely to be a measure of exposure accumulated in preceding months [24, 25]. This is consistent with the findings of this analysis where high clinical incidence rates in under 5 s were associated with an increased risk of a positive RDT in pregnant women for the next 3 months, as well as a recent study demonstrating that in areas of sustained, seasonal transmission a substantial proportion of women attending ANC appointments remain infected throughout the dry season [26]. The models that assumed a non-linear relationship between clinical incidence in under 5 s and ANC prevalence were superior in terms of AIC value and out-of-sample predictive power. The best-fit function produces a curve whereby increasing clinical incidence in children under 5 is approximately linearly associated with larger effects upon ANC prevalence up until around 3 cases per child per year, where it begins to plateau. This shape has been observed in multiple cross-sectional surveys comparing malaria prevalence with clinical incidence [20]. This is likely a product of heterogeneity in mosquito biting (some people are bitten substantially more than others) leading to repeatedly infected people developing asymptomatic infections (so new infections occur in people already infected meaning that there is no change in prevalence).

Due to the changes in the model fit between sites (significantly different *h* parameter values), the model cannot currently be used to predict ANC prevalence from incidence alone. For example, the best fitting model systematically under-predicted the level of ANC prevalence in Walikale, which has similar rates of incidence in children under 5 s as seen in Shamwana but much lower ANC prevalence (Fig. 3). Some of the differences between sites may be accounted for if there was more precise ANC data on factors known to affect the epidemiology of malaria in pregnancy such as timing of gestation [27] and parity. The sensitivity of malaria RDTs are known to vary depending on the number of children that a woman has already had, with more children meaning a likely history of exposure to the parasite during pregnancy and a developed placental immunity [28]. Alternatively, the variation between sites could be attributable to poor incidence estimates at some locations due to sparse health systems, insecurity, inaccurate estimates of population size, or short-term population movement into areas of higher risk (e.g. forested areas). Analysis of mobile phone data in malaria endemic countries shows large-scale population movement within and between countries [29, 30]. The infrequency of national census surveys may therefore limit the accuracy of incidence estimates derived from these surveys. However, census data was only used for two of the sites in the MSF dataset and the incidence recorded at those two sites (Baraka and Kimbi-Lulimba) was not unusual when compared to the other locations. To redress some of the uncertainty in the data, the NENL model was fit using several different maximum lag values (see Additional file 1), with the general results remaining the same for maximum lag values of 2 or 4. However there is still uncertainty in the data that the current model is unable to capture (Fig. 6). The analysis should be repeated as more data become available in order to reduce uncertainty in the model and refine predictions (Additional file 2).

These results have practical implications for the proposed use of ANC prevalence as a tool to monitor malaria. This method has established, at these 5 sites at least, that ANC prevalence seems to be a promising, simple, and cost-effective measure of recent malaria incidence. This has important applications in humanitarian settings and beyond. Good quality population size estimates are difficult, expensive to obtain, and are only available in a small number of sites where MSF operate. ANC data is much more widely available, and this work suggests it should be used to monitor recent trends in malaria endemicity over simple case count data alone. As an illustration of its importance it was unclear from hospital case counts data whether malaria transmission was increasing in sites in Eastern DRC around Baraka or not. Case counts had risen dramatically, though this may have been because of increased investment by MSF (for example the use of mobile malaria teams to diagnose and treat the wider population) or a true increase in disease transmission. The spectrum of mosquitoes resistant to pyrethroid insecticide and the possibility of the spread of drug resistant parasites means that local control interventions need to monitor secular trends in transmission regularly and tailor their programmes to maintain good levels of control. Examination of ANC data in these sites during this period would have provided a simple, unbiased method of raising concerns over recent increases in transmission. This method also provides a way of singling out changes in incidence that should be matched by a corresponding change in ANC prevalence, but this does not happen. For example, a change in reporting capacity or surveillance may induce an increase in incidence, but this would then not be followed by an increase in ANC prevalence so those responsible for monitoring malaria can be confident that the increase in incidence was not due to increase in overall transmission.

Humanitarian organizations and other bodies are regularly trialling new methods of malaria control in specific areas to try and meet local needs. For example, MSF have used mobile malaria teams, community-based malaria management and different models of health centre support in different areas of the DRC. The evidence-base to support these interventions is lacking due to the huge expense and infeasibility of conducting large RCTs in some areas. The full effect of a sustained decrease in transmission due to an intervention may not be observable in ANC prevalence measurements until several months after it begins, therefore availability of routine ANC data from a strategy of IST alongside IPTp in area where the intervention is introduced, combined with the model outlined here, could provide a low-cost measure of triaging new interventions to see which should go on for more thorough investigation.

ANC prevalence was found not to be useful for predicting future incidence in children under 5 years old, so there is no evidence to support its use in predicting future malaria trends from this work. However, it may be that combining ANC prevalence with other data such as the amount of rainfall may allow for models with better predictive power, though this analysis is beyond the scope of this work. In the future, it would be beneficial to invert the relationship used in this work to use ANC prevalence to predict past trends in incidence, useful in many of humanitarian contexts discussed where cases or denominator populations cannot be reliably recorded.

## Conclusions

This work found that time-series data of clinical incidence in children under 5 years predicts future prevalence of infection in pregnant women, but not the other way around. Increases in clinical incidence were associated with increased risk of a positive RDT in a pregnant woman for the next 3 months, with the opposite being true for decreases in incidence. This helps us to understand the role that ANC prevalence can play as a tool for retrospectively examining how malaria transmission has changed in a location over time. Though ANC prevalence derived from routinely collected clinical data may not directly reflect clinical incidence rates calculated from accurate population data, this analysis establishes that it does correspond to recent trends in malaria transmission and provides a simple to collect metric in situations where good malaria data is sparse, such as chaotic, rapidly changing humanitarian crises.

## Additional files

**Additional file 1: Figure S1.** A table of the values of four information criteria for different lag orders, used to determine the lag order of the VAR model. **Figures S2**–**S4.** Copies of Fig. 4 whereby the NENL model is fitted to data using a lag order of 1, 2, or 4 months. **Figure S5.** A copy of Fig. 4 using the NELL model described in the analysis rather than the NENL model.

**Additional file 2.** This dataset contains monthly time series data for all 5 MSF locations, including ANC visits, ANC prevalence, and clinical incidence in children under 5 years old.

## Abbreviations

ANC: ante natal care
AIC: Akaike information criterion
ACT: artemisinin-based combination therapy
SP: sulfadoxine-pyrimethamine
MSF: Médecins Sans Frontières
RDT: rapid diagnostic test
IST: intermittent screening and treatment
IDP: internally displaced persons
IPTp: intermittent preventative treatment during pregnancy
DRC: Democratic Republic of Congo
DLNM: distributed lag non-linear models
VAR: vector autoregressive model
