# Supplementary material for: Using ante-natal clinic prevalence data to monitor temporal changes in malaria incidence in a humanitarian setting in the Democratic Republic of Congo
Source: Malar J. 2018 Aug 29;17:312. doi: 10.1186/s12936-018-2460-9 (PMC6114784; doi:10.1186/s12936-018-2460-9)

**Additional file**

**Using ante-natal clinic prevalence data to monitor temporal changes in malaria incidence in a humanitarian setting in the Democratic Republic of Congo**

Joel Hellewell^1^^ (j.hellewell14@imperial.ac.uk), Patrick Walker^1^ (patrick.walker06@imperial.ac.uk), Azra Ghani^1^ (a.ghani@imperial.ac.uk), Bhargavi Rao^2*^ (bhargavi.rao@london.msf.org), Thomas S. Churcher^1*^ (thomas.churcher@imperial.ac.uk)

^1^ MRC Centre for Outbreak Analysis and Modelling, Imperial College London, London, United Kingdom.

^2^ Manson Unit, Médecins Sans Frontières (Operational Centre Amsterdam), London, United Kingdon.

^^^Corresponding author

^*^T.C. and B.R. are shared last authorship

**Table S1: A table of the values for 4 different information criterion when fitting VAR models with different lag orders. In the manuscript AIC is used to choose a lag order of 3. Below the DLNM model “NENL” is fit to different lag orders to show how the choice of lag order affects the results presented in the manuscript.**

|  | Lag order = 1 | Lag order = 2 | Lag order = 3 | Lag order = 4 |
| --- | --- | --- | --- | --- |
| Akaike information criterion (AIC) | -6.53649 | -6.55116 | **-6.58123** | -6.57792 |
| Bayesian information criterion (BIC) | **-6.33772** | -6.33085 | -6.29626 | -6.232539 |
| Final prediction error (FPE) | 0.00145 | 0.00143 | **0.001387** | 0.001392 |
| Schwarz criterion (SC) | **-6.09881** | -6.05512 | -6.02684 | -5.96516 |

**Figure S2: A copy of Figure 4 when using 1 month of previous data on the clinical incidence in under 5s to predict future ANC prevalence**


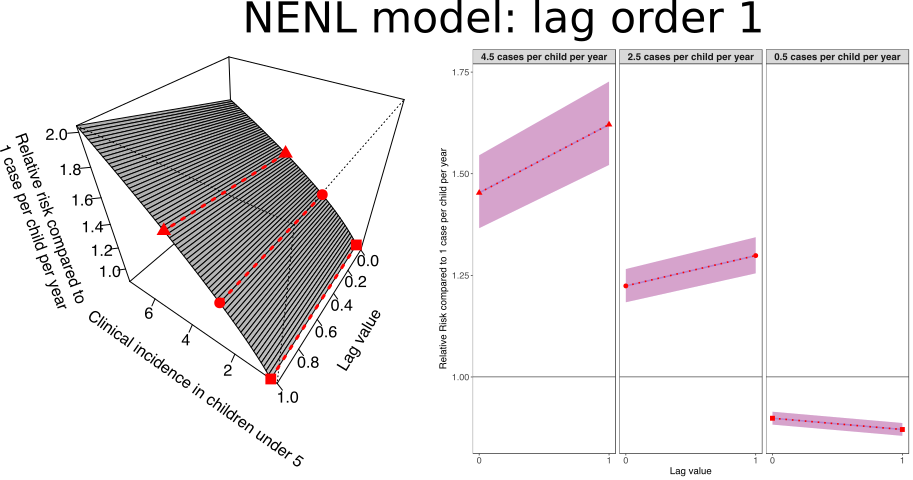


**Figure S3: A copy of Figure 4 when using 2 months of previous data on the clinical incidence in under 5s to predict future ANC prevalence**


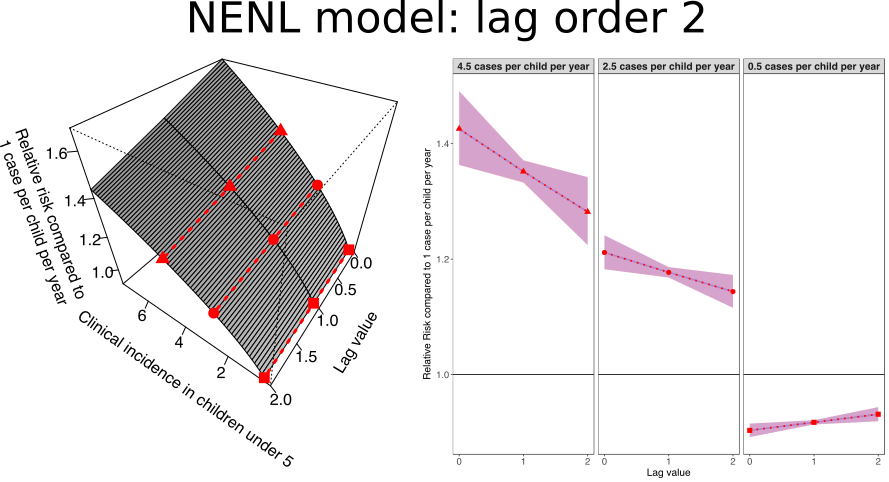


**Figure S4: A copy of Figure 4 when using 4 months of previous data on the clinical incidence in under 5s to predict future ANC prevalence**


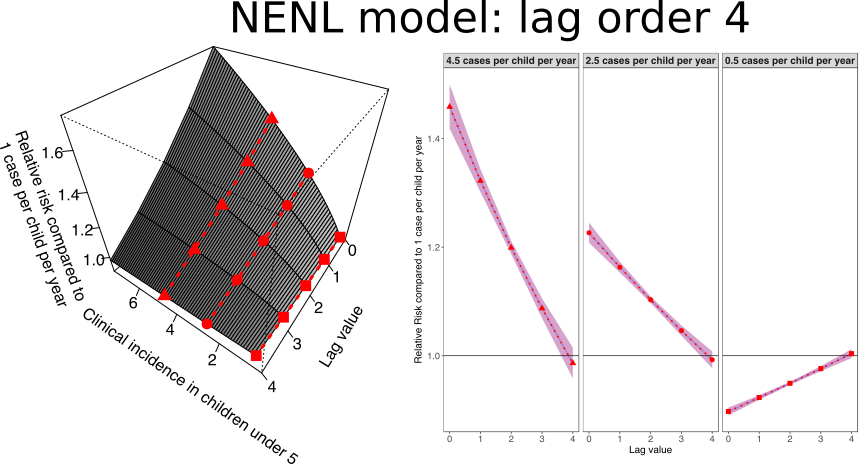


**Figure S5: A copy of figure 4 using the NELL model with maximum lag value of 3**


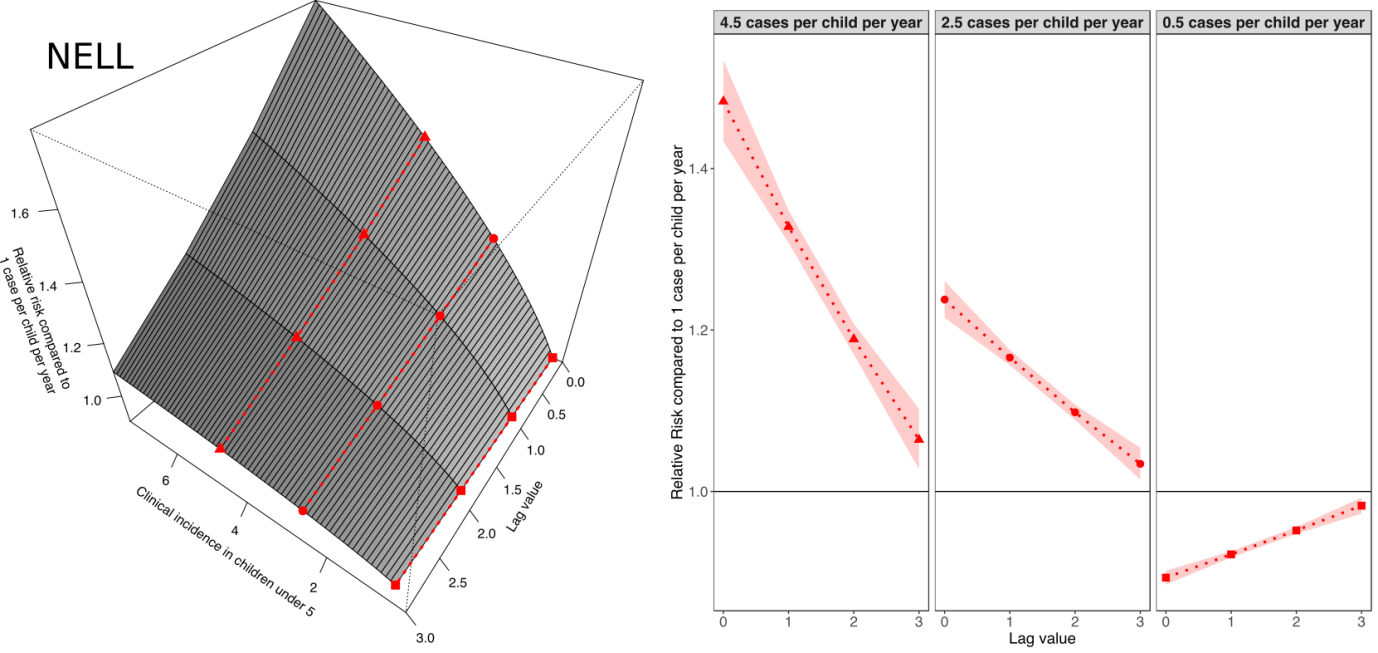

Supplement: Supplementary file 1 — Additional file 1: Figure S1. A table of the values of four information criteria for different lag orders, used to determine the lag order of the VAR model. Figures S2–S4. Copies of Fig. 4 whereby the NENL model is fitted to data using a lag order of 1, 2, or 4 months. Figure S5. A copy of Fig. 4 using the NELL model described in the analysis rather than the NENL model. [file 12936_2018_2460_MOESM1_ESM.docx]
